# RGS4-Deficiency Alters Intracellular Calcium and PKA-Mediated Control of Insulin Secretion in Glucose-Stimulated Beta Islets

**DOI:** 10.3390/biomedicines9081008

**Published:** 2021-08-13

**Authors:** Guillaume Bastin, Lemieux Luu, Battsetseg Batchuluun, Alexandra Mighiu, Stephanie Beadman, Hangjung Zhang, Changhao He, Dana Al Rijjal, Michael B. Wheeler, Scott P. Heximer

**Affiliations:** 1 Department of Physiology, University of Toronto, Toronto, ON M5S 1A8, Canada; lemieux.luu@gmail.com (L.L.); battsetseg.batchuluun@utoronto.ca (B.B.); sorana.mighiu@gmail.com (A.M.); steph.beadman@gmail.com (S.B.); hangjun.zhang@utoronto.ca (H.Z.); chh4002@med.cornell.edu (C.H.); dana.alrijjal@mail.utoronto.ca (D.A.R.); michael.wheeler@utoronto.ca (M.B.W.); scott.heximer@utoronto.ca (S.P.H.); 2 Ted Rogers Centre for Heart Research, Translational Biology and Engineering Program, University of Toronto, Toronto, ON M5G 1M1, Canada; 3 Heart and Stroke/Richard Lewar Centre of Excellence in Cardiovascular Research, Toronto, ON M5S 3H2, Canada

**Keywords:** RGS proteins, glucose-stimulated insulin secretion, intracellular Ca^2+^, cAMP

## Abstract

A number of diverse G-protein signaling pathways have been shown to regulate insulin secretion from pancreatic β-cells. Accordingly, regulator of G-protein signaling (RGS) proteins have also been implicated in coordinating this process. One such protein, RGS4, is reported to show both positive and negative effects on insulin secretion from β-cells depending on the physiologic context under which it was studied. We here use an RGS4-deficient mouse model to characterize previously unknown G-protein signaling pathways that are regulated by RGS4 during glucose-stimulated insulin secretion from the pancreatic islets. Our data show that loss of RGS4 results in a marked deficiency in glucose-stimulated insulin secretion during both phase I and phase II of insulin release in intact mice and isolated islets. These deficiencies are associated with lower cAMP/PKA activity and a loss of normal calcium surge (phase I) and oscillatory (phase II) kinetics behavior in the RGS4-deficient β-cells, suggesting RGS4 may be important for regulation of both Gαi and Gαq signaling control during glucose-stimulated insulin secretion. Together, these studies add to the known list of G-protein coupled signaling events that are controlled by RGS4 during glucose-stimulated insulin secretion and highlight the importance of maintaining normal levels of RGS4 function in healthy pancreatic tissues.

## 1. Introduction

Insulin produced in pancreatic β-cells controls a number of important physiologic processes, including glucose homeostasis [1], cellular respiration [2], feeding behavior [3], tissue differentiation [4,5], and numerous others [6,7]. Because of its primary role in maintaining metabolic homeostasis, insulin secretion is coupled to a wide number of metabolic factors, including glucose [8], fatty acids [9], and amino acids [10]. Within the β-cell, uptake and metabolism of glucose via glycolysis, the tricarboxylic acid (TCA) cycle, and oxidative phosphorylation leads to increased concentrations of intracellular ATP and correspondingly higher ATP/ADP ratios in the cytosol [8]. Increasing ATP/ADP ratios inhibit K_ATP_ channels on the plasma membrane of the β-cell leading to depolarization [8,11,12,13] and activation of voltage-gated Ca^2+^ channels to drive increases in intracellular [Ca^2+^] and insulin granule exocytosis [14,15,16,17,18,19,20]. While there is general agreement about the molecular mechanisms that drive the initial burst of glucose-stimulate insulin secretion from β-cells, much less is understood about the physiologic mechanisms controlling the pulsatile insulin secretion patterns following longer-term exposure to high glucose. Notably, such rhythmic pulses in insulin secretion have been linked to slow (120–300 s period) oscillations in intracellular calcium [16,17,18,21,22,23,24,25], and disruption of their rhythm is believed to predict islet dysfunction and pre-diabetic states in animal models [21,22,24,26] including human patients [19,21].

Heterotrimeric G-protein coupled receptor (GPCRs) pathways have been widely implicated in the regulation of insulin secretion [27,28,29,30,31,32,33,34,35]. While numerous GPCRs are expressed on the surface of β-cells, the predominant physiologic pathways impinging on this process are sympathetic and parasympathetic neurotransmitters, as well as incretin the hormones (e.g., GLP-1 and GIP) released from intestinal cells in response to a meal [31,34]. Each of these pathways is mediated by a distinct set of heterotrimeric G-proteins and downstream effector pathways. During the early stages of feeding, even before increases in blood glucose are detected (known as the “cephalic phase”), parasympathetic activity promotes non-metabolic stimulation of insulin release via acetylcholine activation of M3 muscarinic receptors to promote Gαq/11 activation [30], stimulation of Phospholipase Cβ, and production of IP_3_ (Ca^2+^) and DAG (PKC) second messengers [30,36,37]. Sympathetic neurotransmitters epinephrine and norepinephrine function to limit insulin secretion via α2-adrenergic receptor signaling to promote Gαi/o activation and inhibition of plasma membrane-localized adenylyl cyclases [32,38]. By contrast, incretin hormones help amplify insulin secretion following a meal by using GLP-1 and GIP receptors to promote Gαs activation, stimulation of plasma membrane-localized adenylyl cyclases, increased intracellular cAMP and Epac-mediated inhibition of K_ATP_ channels [31,34]. In normal islets, the nature of insulin secretion is dependent on the continuous integration of these different GPCR signaling pathways in the context of the ever-changing physiologic environment to which the β-cells are exposed. Indeed, coordination of Ca^2+^ and cAMP signaling within β-cells has gained recent momentum as a possible strategy for the prevention and treatment of diabetes [18,21,22,23,24,25,39,40,41].

RGS proteins are important physiologic regulators of Gαi/o and Gαq/11 signaling and have been implicated in the regulation of insulin secretion and pancreatic function by a number of different groups. Notably, the data suggest that different RGS protein effects may be highly context-specific, in part depending on the physiologic stimulus and the nature of the receptor signaling pathways that are activated. RGS2 protects pancreatic islets from excessive insulin secretion and apoptosis and plays a role in maintaining healthy pancreatic β-cell mass and function [42]. RGS16 promotes β-cell function through its ability to attenuate somatostatin-mediated signaling effects on insulin secretion and proliferation in intact islets [43]. RGS7, in a complex with the atypical G-protein β subunit Gβ5, promotes both M3 receptor-mediated insulin secretion [44] and glucose-stimulated insulin secretion through regulation of GPCR-dependent PKC activity upstream of insulin granule release [45]. By contrast, RGS4 appears to play a more complicated role in regulating pancreatic function, showing both insulin-promoting and insulin-inhibiting activities through the regulation of different G-protein signaling pathways in β-cells and in its response to pathophysiologic stimuli [28,30].

Specifically, Wess and colleagues reported that relative to other RGS protein family members, RGS4 was highly expressed in both MIN6 insulinoma (β-cell model) lines and murine pancreatic islets [30]. These authors showed RGS4 tempers M3-muscarinic receptor/Gq-mediated stimulation of insulin secretion from MIN6 cells and isolated islet preparations treated with the parasympathetic agonist, OxO-M [30]. Fajas and coworkers also reported that RGS4-deficient animals showed evidence of metabolic dysfunction, including decreased glucose-stimulated insulin secretion in vivo [28], increased circulating FFAs and catecholamines, despite showing normal insulin-releasing function in isolated islet preparations [28]. Given these conflicting data, and the fact that our own previous study had reported pathophysiologic decreases in RGS4 expression following chronic treatment of human pancreatic islets with free fatty acid [46], we set out here to define the role of RGS4 in regulating glucose stimulating insulin-secretion in vitro and in vivo.

## 2. Material and Methods

### 2.1. Material

HEK 201 cells were a kind gift from Zhong-Ping Feng (University of Toronto, Toronto, ON, Canada), Min6 cells were kindly provided by Michael Wheeler (University of Toronto, Toronto, ON, Canada), RGS4-GFP mice were a generous gift from Pat Levitt (University of Southern California, USA), RGS4KO mice were published previously [47,48,49], and related information are available at https://www.jax.org/strain/005833 (accessed on 1 August 2021). All other compounds were from Sigma (Mississauga, ON, Canada) unless otherwise stated.

### 2.2. OGTT

The oral glucose tolerance test (OGTT) was performed as previously published [50,51]. Briefly, littermate adult mice were fasted overnight prior to the test. At *t* = 0 min, 2 g/kg glucose was administered by oral gavage, and tail vein blood samples were collected at 0, 10, 20, 30, 45, 60, and 120 min post-gavage. Glycemia was measured using a glucometer (Bayer, Leverkusen, Germany). Blood serum was separated using centrifugation, and insulin was quantified from each by duplicate ELISA assays (Crystal Chem, Chicago, IL, USA).

### 2.3. Isolation and Culture of Pancreatic Islets

The protocol for islet isolation and dispersion was as previously described [52]. Briefly, pancreatic islets were isolated from murine pancreas following collagenase perfusion through the common bile duct. RPMI-1640 containing 10% FBS, 1% penicillin/streptomycin, 1% L-glutamine was used to stop the collagenase activity. Islets were isolated in two successive steps to maximize debris removal before use in the experiments described below. For GSIS experiments, islets were allowed to rest overnight in RPMI-1640 containing 10% FBS, 1% penicillin/streptomycin, 1% L-glutamine in a humid 37 °C chamber with 5% CO_2_.

### 2.4. Secreted and Total Insulin Quantitation

GSIS and insulin content quantification was described previously [52]. Rested islets were incubated in Krebs-Ringer Bicarbonate (KRB): 115 mM NaCl, 5 mM KCl, 24 mM NaHCO_3_, 2.5 mM CaCl_2_, 1 mM MgCl_2_,10 mM HEPES, containing 2% bovine serum albumin, 1 mM glucose for 1 h in a humidified chamber at 37 °C at 5% CO_2_. Islets of similar size were aliquoted into 1.5 mL tubes (10 islets per tube). Islets were suspended in 500 µL KRB containing 5.6 mM glucose and then 20 mM glucose for 30 min in a humid chamber at 37 °C containing 5% CO_2_ before the supernatants were collected to quantify insulin secretion at both low and high glucose concentrations. Islets were then lysed for DNA quantification to normalize secreted insulin amounts to cell numbers. Each sample condition was performed in triplicate, and insulin was quantified in duplicate for each sample using an HTRF kit according to the manufacturer’s (Cisbio, Bedford, MA, USA) instructions. Total islet insulin content was quantified as described above from total islet lysates following two freeze-thaw cycles. Drugs for GSIS experiments were added at the indicated concentrations to both 5.6 mM and 20 mM KRB solutions, so they were present throughout the experiment with the exception of rapamycin (50 nM) and pertussis toxin (0.2 µg/mL) that were added 1 hr and 16 hrs prior to the start of the experiment, respectively.

### 2.5. RT-qPCR

Total RNA isolation, RT-qPCR, and quantitation of various RGS protein mRNAs were performed as previously described [53,54]. Other primers sequences are as following: Hif1α forward, 5′-TCAAGTCAGCAACGTGGAAG-3′, reverse, 5′-TATCGAGGCTGTGTCGACYG-3′; LC3 forward, 5′-CTGCTCTGTCTTGTG TAGGT-3′, reverse, 5′-TGTGTGCCTTTATTAGTGCATC-3′; UVRAG forward, 5′-CTACCTGGATGGGCTGAAGT-3′, reverse, 5′-TGCGAACACAGTTCTGGT CC-3′; ATG9 forward, 5′-CACTTCAATGAGCTGGAGCA-3′, reverse, 5′-GTG AGGACGTGTTCCACAGC-3′; GLUT-1 forward, 5′-TCAACACGGCCTTCA CTG-3′, reverse, 5′-CACGATGCTCAGATAGGACATC-3′; and Hexokinase-II forward, 5′-TGATCGCCTGCTTATTCACGG-3′ Reverse 5′-AACCGCCTAGAA ATCTCCAGA-3′. mRNA quantification was performed in duplicate (1 mouse/sample) and carried out on three mice per RGS4-WT and -KO genotypes.

### 2.6. Confocal Microscopy

Wild-type and RGS4-GFP islets were imaged by confocal microscopy, performed on live tissues at 37 °C in an environmental chamber maintained at 5% CO_2_ coupled to an Olympus FluoViewTM FV1000 laser-scanning confocal microscope. Images were captured from an equatorial slice of the islets using a 20× objective with 0.7 numerical aperture. Image acquisition and processing software were as described previously [49].

### 2.7. Electron Microscopy

Islets were prepared and imaged as described previously [55]. Dense core and light core (grey) granules corresponding to mature and immature insulin granules, defined from previously published [56], were manually counted and quantified with the experimenter blinded to the condition. The distance of each insulin granule to the respective β-cell plasma membrane was measured using ImageJ.

### 2.8. Immunohistochemistry

Histology sample preparation was essentially as described previously [57]. Murine whole pancreata were extracted and fixed in 10% neutral buffered formalin. Four μm formalin-fixed paraffin-embedded sections were de-waxed and rehydrated to prepare for immunohistochemistry. Endogenous peroxidase and biotin activities were blocked using 3% hydrogen peroxide and avidin/biotin blocking kit (Fisher Scientific Cat# TA-015-BB; Ilkirch, France), respectively, prior to addition of 10% normal serum (from the species where the secondary antibody is obtained) blocking for 10 min. Sections were incubated at room temperature with the appropriate primary antibodies (anti-glucagon 1:300 Sigma; anti-insulin 1:100 Invitrogen, Waltham, MA, USA). Following repeated washing steps, biotinylated secondary antibodies (Vector Labs, Burlingame, CA, USA) were applied for 30 min at room temperature before washing and addition of horseradish peroxidase-conjugated ultrastreptavidin labeling reagent (Biolegend, San Diego, CA, USA). Following color development and washing in TBS (Tris-buffered saline), tissue sections were counterstained lightly with Mayer’s Hematoxylin, dehydrated in alcohols, cleared in xylene, and mounted in Permount (Fisher, cat# SP15-500; Waltham, MA, USA). Histology images were acquired with Imagescope (v.12.3.2.8013; Leica Biosystems, Wetzlar, Germany) and viewed with NDP view.2 software (Hamamatsu Photonics, Hamamatsu City, Japan).

### 2.9. Islet Dispersion and Intracellular Calcium Imaging

The detailed protocol for islet dispersion was as published previously [52]. Isolated islets were digested in Dispase-II (Roche Applied Science, Penzberg, Germany) for 6 min at 37 °C, followed by gentle trituration. Digestion was stopped by the addition of RPMI-1640 media containing 10% fetal bovine serum. Dispersed cells were washed and plated onto glass coverslips coated with poly-l-lysine and cultured at 37 °C. Real-time intracellular calcium imaging was performed on dispersed β-cells as described [53]. Briefly, dispersed islets β-cells were loaded with Fura-2, incubated in a perfusion chamber, and selected as ROIs (regions of interest) to monitor for changes in intracellular calcium dynamics over a 30-min experiment. 300 ms exposures were taken every 6 s, using a BX51W1 microscope (Olympus, Tokyo, Japan) with a 20/0.95 water immersion objective and cooled charge-coupled device camera. Dual excitation at 340/380 nm was used, and emission at 510 nm was recorded (ImageMaster 3 software, Photon Technology International, London, ON, Canada). The perfusate was applied using a multichannel port at a flow of 1 mL/min to allow switching from low to high glucose KRB and KCl containing solutions.

### 2.10. Animal Care

All animal work was conducted under guidelines established by the Canadian Council on Animal Care (CCAC). All experimental protocols were approved by the Institutional Animal Care and Use Committee at the University of Toronto and conducted in accordance with Canadian animal protection laws. The University of Toronto animal use protocol (AUP) # is 20011793. Expiry date 15 January 2022. All animals were housed under a standard 14:10 h light-dark cycle with ad libitum access to water and chow, notwithstanding experimental fasting protocols as required.

### 2.11. Choice of Sexual Genders

A mixture of male and female adult C57Bl/6 mice was used for the studies described below. Historic data from our group predict higher variability in the glycemic responses of female compared to male mice following oral glucose challenge, presumably due to inter-subject variations in circulating levels of female sex hormones. Accordingly, potential in vivo phenotypes were identified first in male mice and subsequently examined in mixed gender experiments. Thus, combined data for our in vivo work represent a 2:1 male to female ratio. Based on our experience that varying sex hormone levels would not have the same confounding effects on explanted tissues and cells, a roughly 1:1 mixture of males and females was used for all the remaining ex vivo experiments in this study.

### 2.12. Statistics

The results displayed are all means. Where indicated, Student t-test analysis, one-way, and Two-way ANOVA with Tukey’s post-hoc analysis were used to analyze the experimental results. Fisher’s exact test was used for nonparametric comparisons. * *p* < 0.05 was considered significant. Error bars depict standard errors of the mean (S.E.M.) for all graphs.

## 3. Results

**Figure 1:**
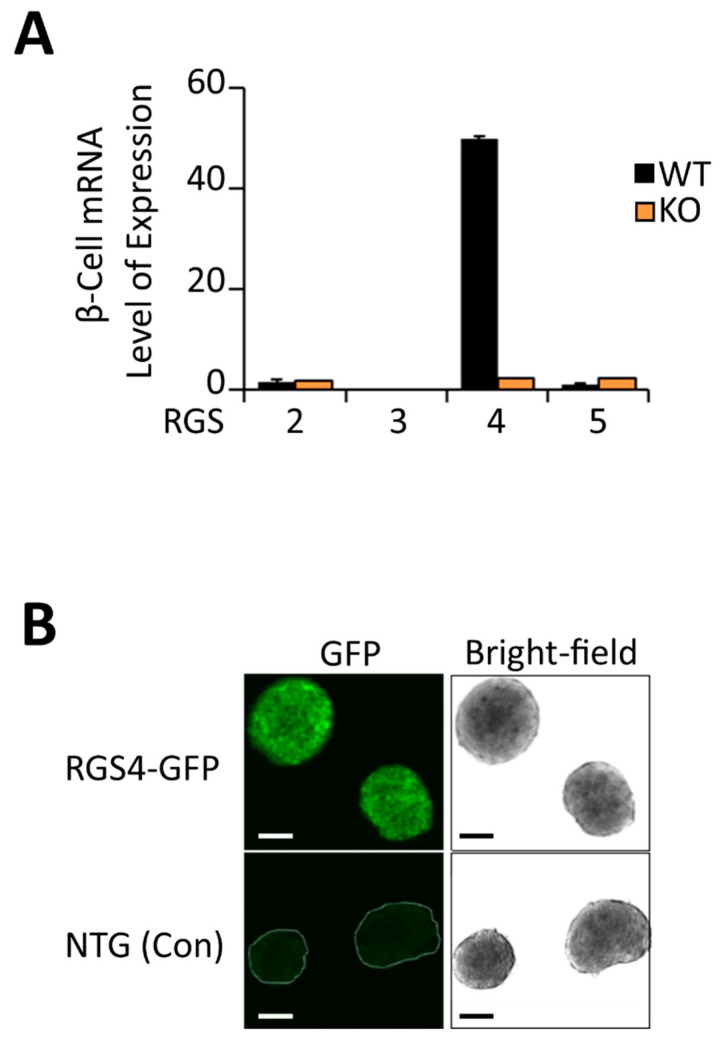
RGS4 is highly expressed in murine pancreatic islets. (**A**) qRT-PCR quantitation of relative RGS protein mRNA expression in wild-type and RGS4-deficient murine pancreatic islets. Data are normalized to 18S ribosomal RNA. (**B**) Bright-field and fluorescent confocal microscopy images of pancreatic islets from wild-type (non-transgenic control) and RGS4-GFP mice. Scale bars denote 100 µm.

Real-time RT-PCR analysis showed RGS4 was expressed at much higher levels in murine pancreatic islets (50-fold higher) levels than other RGS proteins in the R4/B subgroup (Figure 1A). Confocal microscopy of islets isolated from RGS4-GFP reporter mice [58] nicely corroborated this point (Figure 1B). Despite overlapping biochemical selectivity, no compensatory mRNA expression changes were observed between the other RGS proteins examined, suggesting a potentially unique function for RGS4 in pancreatic islets.

**Figure 2:**
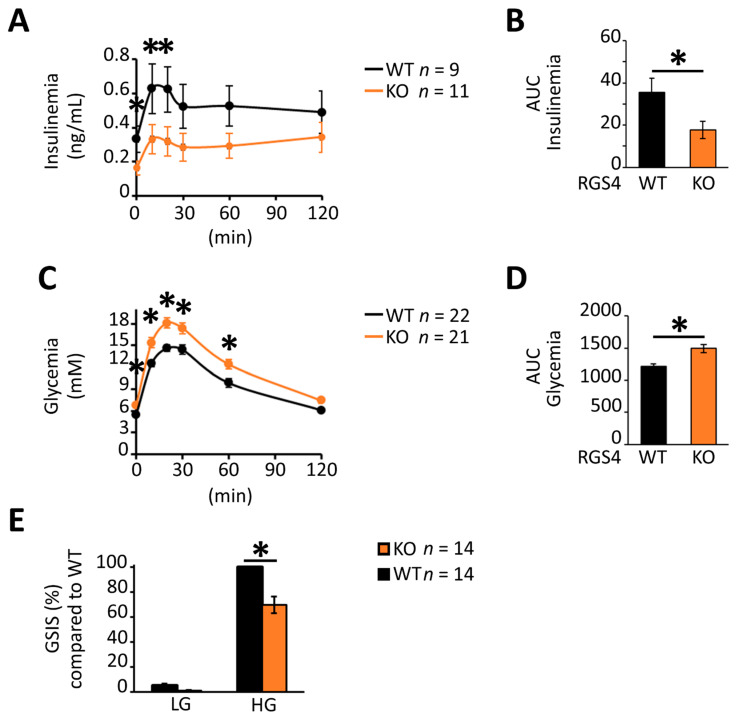
RGS-deficient islets show decreased glucose-stimulated insulin secretion (GSIS) in vivo and in vitro. (**A**–**D**) Kinetic profiles and corresponding area under the curve (AUC) data for serum insulin (**A**,**B**) and glycemia levels (**C**,**D**) following oral glucose challenge of fasted WT and RGS4-deficient mice. Panels **C** and **D** correspond to combined analysis of ~2:1 ratio of male to female mice. (**E**) GSIS on isolated islet preparations. Isolated islets from WT and RGS4-deficient mice were treated with 5.6 mM (low glucose, LG) or 20 mM (high glucose, HG) for 30 min and the total secreted insulin determined from the supernatant. Data are expressed as GSIS% relative to that for WT islets challenged with 20 mM glucose. * *p* < 0.05; [*t*-test], (**C**,**D**) one-way ANOVA and Tukey’s post-hoc test, (**A**,**C**) two-way ANOVA and Tukey’s post-hoc test (**E**).

Oral glucose challenge resulted in lower serum insulin levels in global RGS4 knockout compared to wild-type mice (Figure 2A,B). The glycemic effect was observed in both male and female mice, albeit with a more pronounced difference in the male cohort (Figure S1). Kinetic profiling of oral glucose-stimulated serum insulin levels showed RGS4-deficient mice had lower serum insulin levels during both the early (Phase I) and late (Phase II) phase of the feeding response (Figure 2A, AUC; *p* < 0.05). In agreement with these results, blood glucose levels were higher in RGS4-deficient mice at baseline and following oral glucose challenge (OGTT) compared to those of wild-type mice (Figure 2C,D). To rule out the potential effect of other RGS4-expressing tissues (brain, muscle, etc.) on the OGTT results in our global knockout model, glucose-stimulated insulin secretion (GSIS) studies were performed on isolated islet preparations. Consistent with the results in vivo, RGS4-deficient islets showed a 30% decrease in GSIS compared to wild-type (Figure 2E). Together these data suggest an important role for RGS4 as a positive regulator of glucose-stimulated insulin secretion and serum plasma accretion in isolated islets and intact animals, respectively.

**Figure 3:**
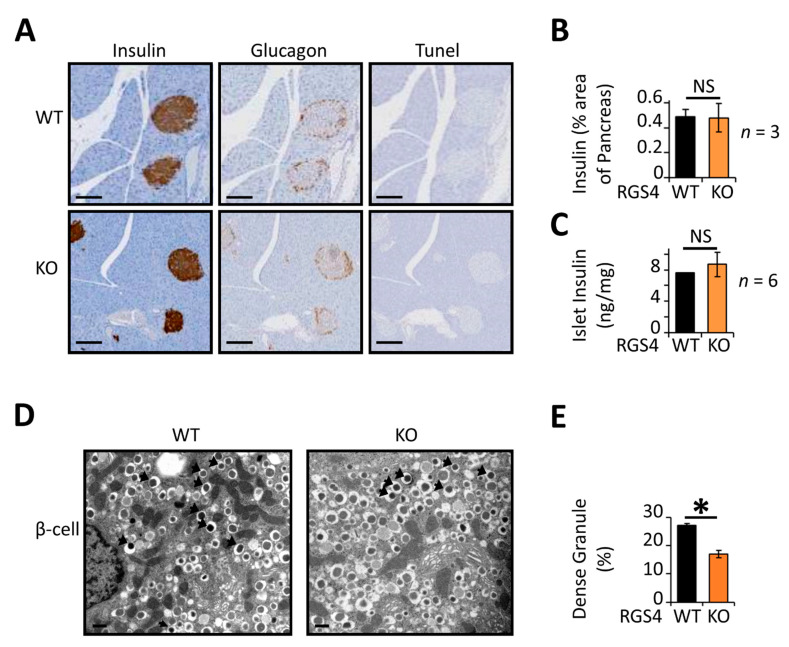
RGS4-deficient pancreatic islets contain relatively low mature insulin granule content. (**A**) Representative images of pancreatic sections from mice stained for insulin, glucagon, and apoptotic indices (Tunel). Scale bar, 100 µm. (**B**) Average islet area was determined from pancreatic sections using three distinct sections from each animal and 3 mice per genotype. (**C**) Total insulin content within isolated islet preparations was normalized to pancreas mass. (**D**) Representative electron microscopy images of WT and RGS4-deficient β-cells. Black arrows point toward dense (mature) granules of insulin. Scale bar, 0.3 µm. (**E**) Relative proportion of dense granules is lower in RGS4-deficient compared to WT mice; these data have been determined from analysis of 6426 for WT and 9501 granules for KO. Experimenters were blinded to sample genotype. * *p* < 0.05; NS, not significant [*t*-test (**B**,**C**,**E**)].

Histologic studies revealed no abnormalities in islet morphology or apoptotic (TUNEL-staining) indices (Figure 3A). Immunostaining for insulin and glucagon revealed similar islet architecture between genotypes. Specifically, the organization of insulin-containing cells on the “inner area” of the islet and glucagon-containing cells on the “outer crown” was similar for RGS4-KO and wild-type mice. The ratio of insulin-containing cell area to pancreatic tissue area was also similar between RGS4-deficient and wild-type samples (Figure 3B). Consistent with these results, the total insulin peptide content was similar between isolated islet preparations from RGS4-deficient and wild-type mice (Figure 3C). High-resolution images collected by transmission electron microscopy (TEM) revealed no differences in the relative distribution of insulin granules between the readily releasable and reserve granule pools (Figure S2). However, it was interesting to observe that the proportion of mature insulin granules, typified by a dense electron impermeable core, was reduced from 27% in wild-type β-cells to 17% in those from RGS4-deficient mice (Figure 3D,E).

**Figure 4:**
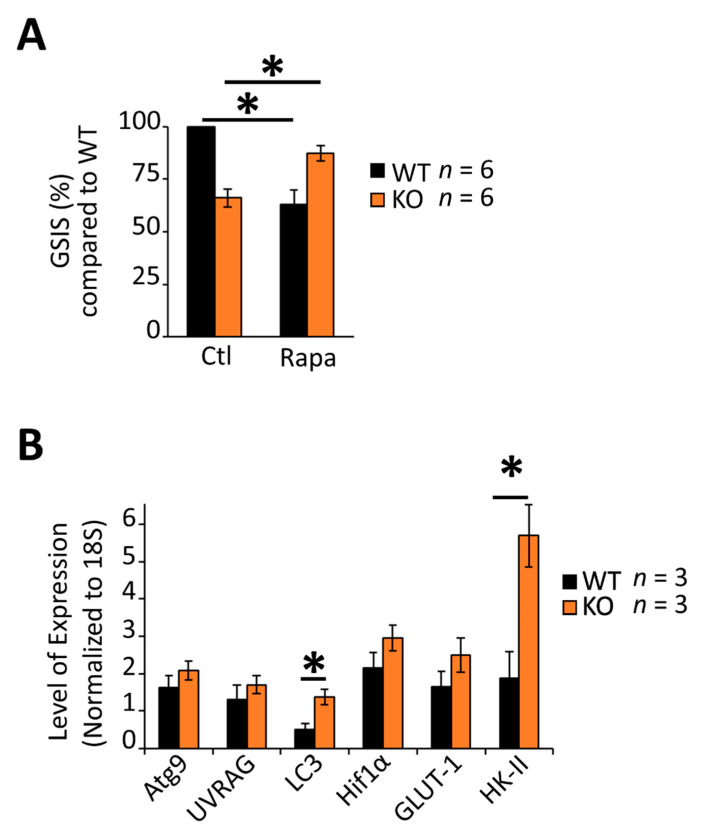
Altered glucose responsiveness is associated with dysregulation of the MTOR-C1 pathway and glucose homeostasis gene expression in RGS4-deficient islets. (**A**) Glucose-stimulated insulin secretion (GSIS) was measured in isolated islets from WT and RGS4-KO mice following 30 min glucose challenge (20 mM) with and without the addition of the MTORC1 inhibitor rapamycin (50 nM). Data are expressed as GSIS% relative to that for WT islets. (**B**) qRT-PCR analysis of mRNA expression of select genes related to autophagic or glucose homeostasis in WT and RGS4-deficient isolated islets. * *p* < 0.05; [one-way ANOVA and Tukey’s post-hoc test (**A**,**B**)].

β-cells use MTORC1 activity to coordinate nascent insulin secretory granule homeostasis (synthesis and degradation) under both nutrient-starved and fed conditions [59]. Consistent with data from multiple laboratories [59,60,61], treatment of isolated wild-type islets with the MTORC1 inhibitor, rapamycin, decreased glucose-stimulated insulin secretion by roughly 30% (Figure 4A), suggesting that MTORC1 activity was important for the normal physiologic response to glucose. By contrast, however, rapamycin failed to decrease glucose-stimulated insulin secretion in RGS4-deficient islets suggesting that MTORC1 activity may be lower in knockout islets. mRNA expression of several upstream and downstream factors in the glucose/glycolysis-mTORC1-autophagy axis [62,63,64] were consistent with MTORC1 inhibition (e.g., increased hexokinase II (HK2)) [65,66] and upregulation of autophagic signaling pathways (increased LC3) in the knockout cells (Figure 4B).

**Figure 5:**
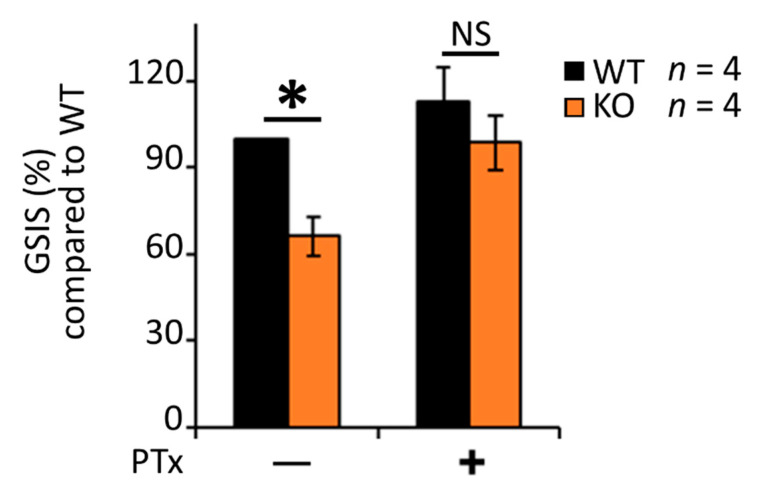
Pertussis toxin (PTx) largely corrects the glucose-stimulated insulin secretion defect in RGS4-deficient islets. Glucose-stimulated insulin secretion (GSIS) was measured in isolated islets from WT and RGS4-KO mice following 30 min glucose challenge (20 mM) with and without the addition of 0.2 µg/mL PTx. Data are expressed as GSIS% relative to that for WT islets. * *p* < 0.05; NS, not significant [one-way ANOVA and Tukey’s post-hoc test].

Gαi/o inhibition using *Bordella pertussis* toxin (PTx) is known to enhance insulin secretion from β-cells [27,43,67,68,69]. We asked whether RGS4′s role in maintaining glucose stimulated insulin secretion may also be linked to its biochemical activity as a Gαi/o inhibitor. Indeed, the GSIS deficiency in RGS4KO islets was largely rescued by PTx treatment (Figure 5). Since RGS16 was reported to inhibit Gαi/o-coupled somatostatin receptor (SSTR) activity [43] from promoting GSIS, we wondered whether enhanced SSTR signaling might explain the decreased GSIS we observed in isolated islets from RGS4-deficient mice. A pan-inhibitor of SSTR function (cyclo-SST) modestly increased GSIS in wild-type islets but was not able to rescue any insulin secretion defect in RGS4-deficient islets (Figure S3). These data argued against enhanced SSTR signaling as a primary contributor to the reduced insulin secretion phenotype in our model.

**Figure 6:**
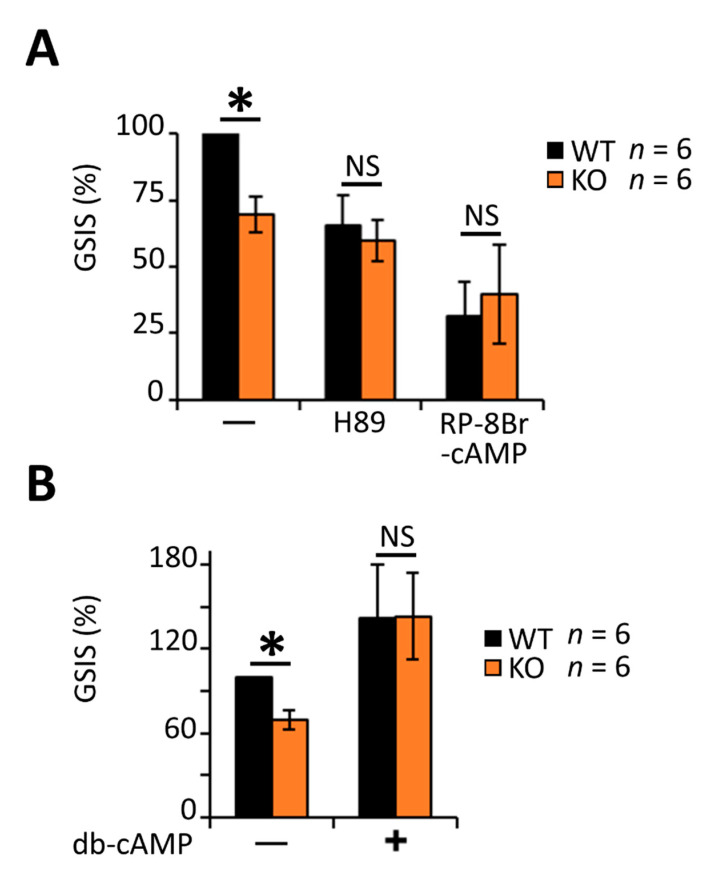
Decreased glucose-stimulated insulin secretion in RGS4-deficient islets is associated with dysregulated cAMP/PKA signaling. Glucose-stimulated insulin secretion (GSIS) was measured in isolated islets from WT and RGS4-KO mice following 30 min glucose challenge (20 mM) with and without the addition of 10 µM of H89, 1 mM of RP-8Br-cAMP (**A**) or 1 mM of db-cAMP (**B**). Data are expressed as GSIS% relative to that for WT islets. * *p* < 0.05; NS, not significant [one-way ANOVA and Tukey’s post-hoc test (**A**,**B**)].

Alternatively, RGS4 was also shown previously to block Gαi/o mediated inhibition of cAMP production [70] to promote increased intracellular cAMP levels. The addition of the PKA inhibitors H89 and Rp-8-Br-cAMPS to cultured islets reduced their insulin secretion following glucose stimulation by 40% and 70%, respectively, in wild-type islets and normalized the differences between wild-type and RGSKO tissues (Figure 6A). By contrast, the addition of a cell-permeable cAMP mimetic, dibutyryl-cAMP (db-cAMP), markedly increased insulin secretion in both wild-type and RGS4-KO islets (Figure 6B). Notably, the effect of db-cAMP was greater on RGS4KO islets to the extent that it normalized the difference in insulin secretion between the two genotypes. Together, these data support a model where RGS4-deficiency leads to altered cAMP signaling and insulin granule maturation/secretion in pancreatic islets.

**Figure 7:**
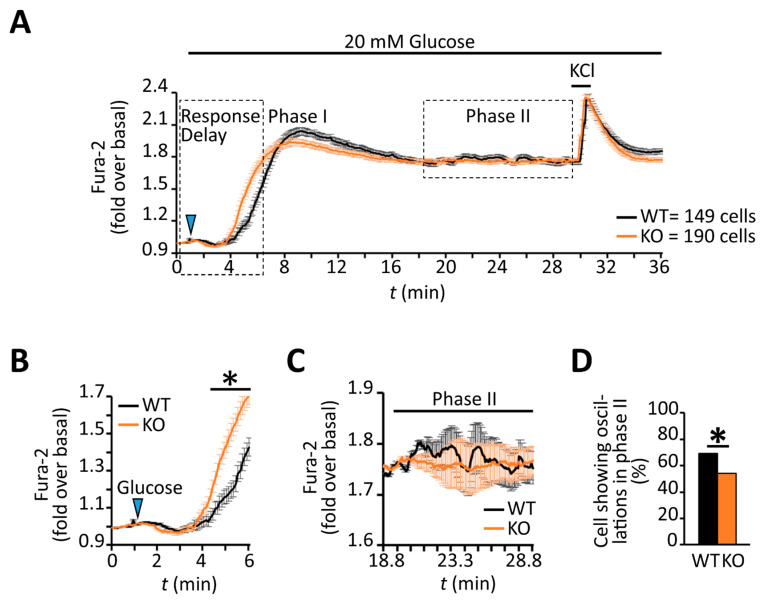
RGS4-deficient β-cells show altered intracellular calcium responses to glucose stimulation. (**A**) Kinetic profile of the glucose-stimulated intracellular calcium response was recorded by fluorescent ratiometric imaging of dispersed β-cells from WT and RGS4-KO pancreata loaded with Fura-2 calcium indicator dye. Panel (**B**) highlights the intracellular calcium kinetics in the early phase (phase I) of the response to glucose challenge. Panel (**C**) highlights the intracellular calcium dynamics in the later phase (phase II) response to glucose, a phase normally associated with oscillatory intracellular calcium and insulin secretion behavior. (**D**) Relative quantitation of the percentage of β-cells showing phase II intracellular calcium oscillations in WT and RGS4-KO mice. * *p* < 0.05; [*t*-test (*B*), Fisher test (**D**)].

cAMP and its effector pathways are implicated in the coordination of both insulin granule secretion and β-cell calcium dynamics [12,22,23,71]. Indeed, PKA inhibition in MIN6 cells was shown to decrease the time required for glucose to produce a rise in intracellular calcium [22]. Consistent with lower levels of PKA activity in RGS4-deficient β-cells, they showed a reduced time interval between glucose addition and the corresponding rise in calcium compared to wild-type cells (Figure 7A,B). Moreover, the average amplitude of the initial calcium peak was lower in RGS4-deficient cells, consistent with altered calcium signaling during the early (0–17 min, Phase I) stage of glucose-stimulated insulin secretion (Figure 7A). Rapid KCl-mediated depolarization of cells at the termination of each experiment revealed similar activation of voltage-gated calcium channels to a strong depolarizing stimulus in both wild-type and knockout cells (Figure 7A).

**Figure 8:**
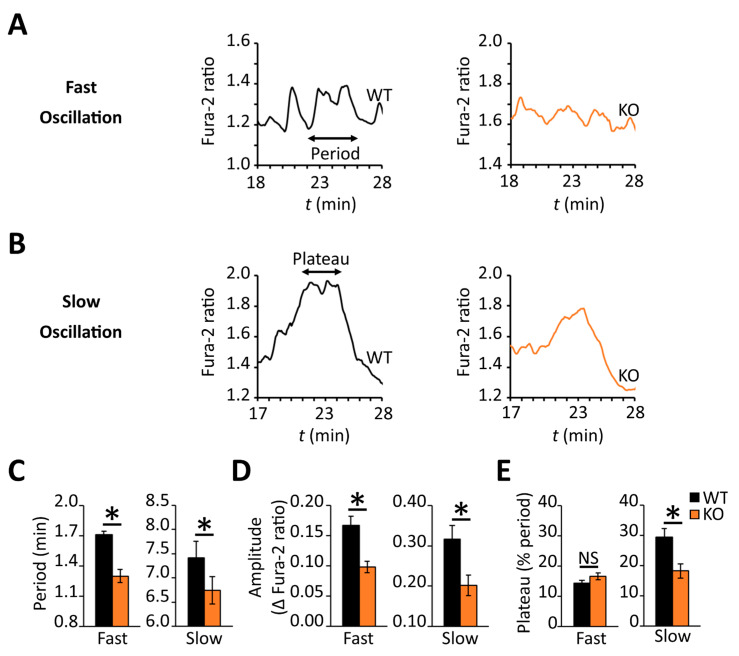
RGS4 promotes increased oscillatory intracellular calcium behavior in dispersed β-cells challenged with glucose. Representative traces of fast-type (**A**) and slow-type (**B**) intracellular calcium oscillations recorded during phase II of the glucose response in dispersed β-cells from WT and RGS4-KO pancreata as in Figure 7 above. The characteristics of the oscillatory behavior for slow and fast oscillations are recorded in panels (**C**), for the period, (**D**) for amplitude, and (**E**), for relative duration of a sustained plateau. In all panels, the data are representative of 180 and 128 fast oscillations and 25 and 48 slow oscillations for WT and KO, respectively. * *p* < 0.05; [*t*-test was applied in panel (**C**–**E**)].

Several laboratories have highlighted the importance of coordinated oscillations of intracellular calcium and cAMP for promoting pulsatile insulin secretion following glucose stimulation [22,23,24,25] during the sustained phase of the response. Although the later stage profile for intracellular calcium (i.e., 17–30 min post-stimulus, Phase II) looked similar across the two β-cell populations (Figure 7A), the wild-type profile showed greater oscillatory activity during this period (Figure 7C). This observation prompted us to characterize the calcium dynamics in individual β-cells. Indeed, a lower percentage of RGS4-deficient β-cells showed oscillatory calcium behavior during the later phase compared to wild-type (54% compared to 69% of all cells measured respectively, *p* < 0.0067) (Figure 7D). Other laboratories have defined different types of late-phase calcium oscillations in glucose-stimulated β-cells based on their frequency and period [16,18,21,22,23,24]. We herein classify three different types of oscillations as fast (<2.5 min period), intermediate (2.5–5.0 min period), or slow (>5.0 min period). Consistent with the summary data above, the analysis revealed that both fast and slow oscillations in RGS4-deficient cells had shortened periods and decreased amplitudes (Figure 8A–D). Lastly, within the subgroup of cells showing slow oscillations, the relative duration of sustained calcium peak plateaus was reduced in RGS4-deficient compared to wild-type β-cells (Figure 8E), suggesting less overall intracellular calcium flux was occurring in the knockouts.

## 4. Discussion

Despite the fact that insulin is one of the most widely studied hormones in mammalian physiology, there are numerous aspects of its synthesis in and secretion that remain to be understood in both healthy and diseased conditions. Heterotrimeric G-proteins have been shown to be critical regulators of insulin secretion at multiple levels of pancreatic β-cell function and thus are considered important therapeutic targets for the prevention and treatment of metabolic dysfunction. We here show that RGS4-deficient mice display markedly decreased GSIS in vivo and in isolated pancreatic islet preparations and that these deficiencies are associated with various disruptions in normal cellular signaling. Since RGS4 can inhibit both the Gq/11 and Gi/o subclasses of heterotrimeric G-proteins, it has the potential to intersect with several different signaling pathways controlling insulin metabolism and secretion. Therefore, it is not surprising that studying RGS4-deficiency under different physiologic contexts may reveal markedly different insulin secretion phenotypes [28,30]. This work aims to characterize some of the previously unidentified mechanisms through which disrupted RGS4 function may lead to dysregulation of insulin secretion and to help integrate the findings from several important papers in the field.

It is notable that our data show some important phenotypic differences between previously published works describing the effect of RGS4 deletion in mice on insulin secretion from the Fajas and Wess laboratories [28,30]. For instance, the Fajas group showed glucose intolerance with decreased insulin secretion in addition to other metabolic defects, including hyperlipidemia and enhanced catecholamine secretion [28]. However, normal GSIS results on isolated islets led them to conclude that their insulin secretion defect was secondary to their hyperlipidemia phenotype. By contrast, the Wess group reported that deletion of RGS4 at either the whole body or β-cell-specific level led to enhanced parasympathetic mediated stimulation of insulin secretion via M3 muscarinic receptor/Gq signaling [30]. It is difficult to compare relative pancreas health (b-cell mass, apoptotic index, insulin granule maturation) between these three different models since our work was the only one to include a full histologic work-up. Wess’s group, however, did report that total insulin levels trended lower in RGS4-deficient compared to wild-type islets, which may have impacted their GSIS data since insulin secretion was normalized to total insulin in those assays.

Examination of the genetic strategies and strains used for RGS4 deletion by the different groups may provide some additional insight into potential differences between them. Importantly, both the Fajas and Wess strains delete the RGS domain-containing exons 3–5 but retain the potential to generate intact RGS4 amino-terminal peptides from the RGS4 promoter. RGS protein amino-terminal sequences are known to interact with intracellular loops on receptors as well as different membrane subdomains and might therefore influence the accessibility of other closely related RGS proteins (RGS5 and RGS16) or G-proteins to those domains. Moreover, the extent to which different lines may have retained elements of the 129 strains on which they were generated remains an important unknown, especially when the extent of backcrossing into C57Bl/6 likely varied between the groups. This is particularly important when considering the large difference in mouse sizes between Fajas’ line and our own (32 g versus 26 g, respectively, at 14–16 weeks) and the fact that their strain was the only one to show signs of early metabolic disease, including higher circulating free fatty acids and liver steatosis (visibly pale livers) on regular chow.

Lastly, experimental and study rationale differences may also help to explain some of the apparent discrepancies and results interpretation for the three studies. Firstly, comparing the GSIS protocols for the three groups, it appears that Wess and Fajas may have both performed their GSIS studies on freshly isolated islets, whereas we invoke an overnight “rest” period to allow islet recovery from the stress of the isolation procedure. Comparative analysis of islet “health” at the time of GSIS measures for the three studies would be required to understand the potential impact of this experimental difference. One possible explanation might be, for example, that RGS4 deficiency resulted in constitutive activation of M3R/Gq or another GSIS-promoting pathway, such that there are different states of insulin granule anabolism/maturation under the different GSIS protocols. It is important to highlight, however, that our GSIS results in rested islets were entirely consistent with in vivo data for both glycemia and insulinemia levels in our mouse line. In fact, this entire study was undertaken to help explain the consistently low insulinemia and high glycemic states we observed in vivo across multiple generations in our RGS4-deficient mice. By contrast, Wess’ group report that RGS4-deficiency selectively enhances M3R/Gq signaling, and insulin secretion was characterized primarily in vitro by performing GSIS studies on RGS4-deficient islets treated with exogenous muscarinic agonist. While we agree with Wess’s interpretation of their agonist-evoked GSIS data, we were compelled by our own data to ask whether RGS4 may be regulating other pathways in β-cells in addition to M3R/Gq. Moving forward, it will be important to determine the extent to which endogenous M3R activity (i.e., via parasympathetic acetylcholine) compared to other G-protein signaling pathways can regulate insulin secretion and glycemic levels in vivo for all three of these RGS4-deficient mouse lines.

Indeed, increased/constitutive Gq activity could help explain the elevated autophagy signals and immature insulin granule phenotypes we observed since PLC/diacylglycerol-dependent PKD activation has been shown to promote a switch between lysosome-dependent (SINGD) and macroautophagic degradation of nascent insulin granules in β-cells [59]. It is also possible that constitutive M3R/Gq signaling promotes higher levels of basal insulin secretion in resting islets which could shift granule maturation homeostasis to a less mature state. Lastly, differences in M3R signaling may be an important driver of some of the calcium differences we observe between wild-type and RGS4 deficient cells (Figure 7 and Figure 8). As discussed above, our work points to the dysregulation of multiple G-protein coupled pathways in RGS4-deficient islets responding to a glucose challenge. We thus set out to characterize other RGS4-regulated pathways that may also be important for modulating β-cell responses to a glucose challenge in vivo.

For instance, the fact that PTx treatment partially rescued the GSIS deficiency in RGS4-deficient islets indicated that Gαi/o signaling might also be increased in our model. Histologic analysis shows the RGS4-deficient islets in our mouse line contain normal β-cell volume, total insulin levels and stockpiles of both plasma membrane docked and readily releasable secretory granules. If RGS4 was required to attenuate Gαo-mediated repression of insulin granule docking [67], we would have expected to see fewer insulin granules in close proximity to the membrane. Instead, our data suggest RGS4 may be affecting another effector of Gαi/o activity in β-cells. Indeed, we found multiple indications that dysfunctional cAMP signaling in RGS4-deficient islets may contribute, at least partly, to the aforementioned decreases in glucose-stimulated insulin secretion. Specifically, the ability of both PKA inhibitors and membrane-permeable cAMP mimetics to normalize functional differences between RGS4-deficient and wild-type islets provided strong evidence for dysregulation of these pathways in our models. By contrast, we were somewhat surprised that a nonselective SSTR antagonist did not, at least partially, rescue GSIS in RGS4-deficient islets, especially given the established role of SSTR in this pathway [35] and the reported ability of RGS16 to inhibit SSTR-coupled Gαi/o signaling in isolated islets to help promote insulin secretion [43]. These data are consistent with marked differences in the biochemical specificity for different G-protein/GPCR pathways for different RGS proteins as reported by numerous groups in the field [45,72,73,74,75]. At this stage, however, we cannot rule out the possibility that cell types other than β-cells may also provide paracrine signals (e.g., glucagon or acetylcholine) that could influence the GSIS phenotypes in our whole body RGS4KO mice. Accordingly, the full spectrum Gαi/o-coupled receptors and agonists that mediate the observed deficiencies in cAMP signaling and GSIS in RGS4-deficient islets remain to be identified.

An important biochemical difference between RGS4 and RGS16 protein is the presence of an inhibitory PIP_3_-binding sequence within its G-protein binding domain [74]. Notably, PIP_3_ has been shown to intersperse with Ca^2+^/calmodulin to regulate RGS4 activity in a cyclical fashion required to generate agonist-evoked calcium oscillations in secretory cells [74]. Importantly, high levels of PIP_3_ are produced in β-cells via autocrine insulin signaling [76,77]. It is known that glucose stimulation of β-cells results in an initial burst of intracellular calcium and coordinated insulin release, followed by an extended second phase where synchronized intracellular calcium and cAMP oscillations are coupled to pulsatile insulin release and PIP_3_ elevations [22]. Indeed, such pulsatility of insulin secretion is often missing in human diabetics, where it has been proposed as an important etiologic factor in type II diabetes [18,19]. It may not be a coincidence, therefore, that our previous work showed that RGS4 mRNA was also downregulated in dysfunctional human islets subjected to chronic oleate exposure [46]. Consistent with a potential role for RGS4 in driving phase II oscillatory events, we observed a marked reduction in the frequency and amplitude of short and long period calcium oscillations in β-cells dispersed from RGS4-deficient islets. While the use of a global knockout for these studies does not allow us to rule out contributing effects of non-β-cell types on the insulin secretion defects observed in vivo and in vitro, the observed defects in β-cell Ca^2+^ signaling in the knockouts suggest an important role for RGS4 in the regulation of β-cell-specific G-protein signaling and function. Future studies will be aimed at determining whether similar β-cell signaling defects (altered cAMP/PKA and Ca^2+^ oscillations) contribute to the abnormal regulation of insulin secretion in β-cell specific RGS4KO models.

**Figure 9:**
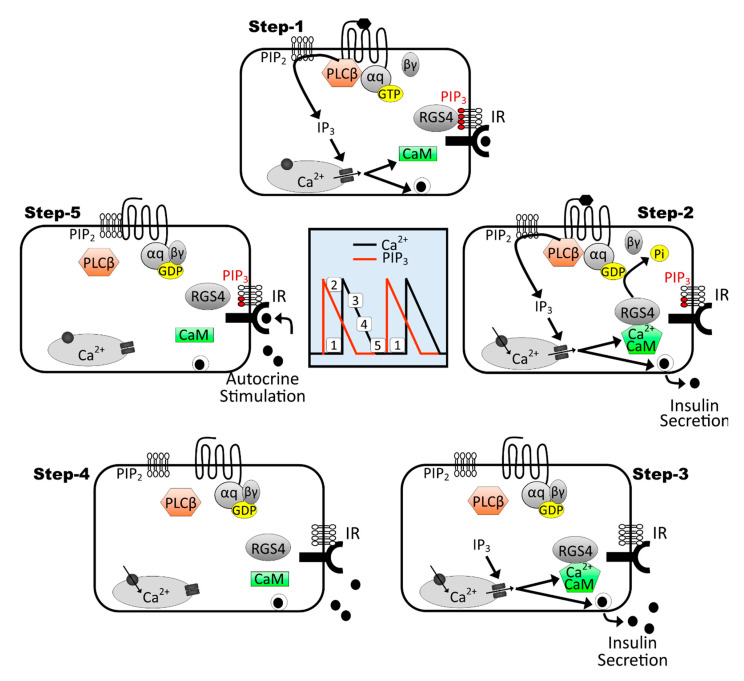
Model for RGS4-mediated control of calcium oscillations in the context of oscillatory insulin receptor-dependent PI3K/PIP3 signaling. This 5-step model extends on previous work from Luo et al., 2001 [78], showing the importance of RGS4/PIP3/Ca^2+^-calmodulin cycling to promote Gq-mediated intracellular calcium oscillations in secretory cells. Central panel shows interspersed timing of intracellular calcium (black trace) and PIP_3_ (red trace) oscillations in a β-cell marked with the relative positions of mapped steps 1–5. Moving clockwise through the steps reveals how the RGS4 inhibition by PIP_3_ and activation by Ca^2+^-calmodulin can lead to oscillatory Gq-mediated calcium oscillations in glucose-stimulated β-cells. Moreover, since intracellular calcium oscillations promote oscillatory insulin secretion, we propose that insulin receptor-dependent PIP_3_ produced via autocrine signaling may contribute to perpetuation of the intracellular Ca^2+^ (and possibly cAMP) cycling in β-cells.

We here propose a multi-step model that extends the work from Wilkie and colleagues [74,78] to explain how RGS4 may drive the oscillatory nature of calcium signaling in pancreatic β-cells (Figure 9). Step 1: endogenous activation of Gq signaling produces intracellular increases in Ca^2+^ and well-characterized insulin pro-secretory effects. Step 2: Ca^2+^/calmodulin formed during peak signaling periods binds RGS4 to help maintain its active/primed state. Step 3: active RGS terminates Gq activity (i.e., terminates both Ca^2+^ oscillations). Step 4: decreasing intracellular calcium promotes dissociation of Ca^2+^/calmodulin from RGS4. Step 5: PIP_3_ produced via autocrine insulin signaling inhibits RGS4 and allows initiation of the next Gq-mediated Ca^2+^ oscillation. It is tempting to speculate that this same model may explain how RGS4 may similarly coordinate cAMP oscillations via its inhibitory activity against Gi. However, reports that cAMP oscillations begin near or at the peak of preceding calcium oscillations [23] may require further interrogation of the relative signaling kinetics and the precise mechanisms that tie these events together. Either way, it appears that loss of RGS4 has a profound impact on both Ca^2+^ and cAMP dynamics in β-cells that can profoundly impair their insulin secretory capacity.

In summary, this work highlights the complexity of G-protein-mediated regulation of glucose-stimulated insulin secretion in β-cells and intact pancreatic islets. RGS proteins provide unique biochemical tools to help understand these complex signaling events. RGS4 is among the most highly expressed RGS superfamily members in β-cells. Since much is already known about its biochemical and physiologic functions, RGS4 remains a key experimental tool to better understand the different G-protein pathways controlling pancreatic function. RGS4 deficiency leads to changes in heterotrimeric G-protein signaling and defective glucose-stimulated insulin secretion in intact mice and isolated islet preparations. Alterations in insulin granule maturation, intracellular Ca^2+^ burst, and oscillatory dynamics, as well as cAMP/PKA signaling, are reported here for the first time as additional consequences to the loss of RGS4 in pancreatic islets. This work further highlights the potential clinical importance of maintaining normal levels of RGS4 function in pre-diabetic and diabetic individuals.

## Data Availability

The data is available upon request.

## Supplementary Materials

The following are available online at https://www.mdpi.com/article/10.3390/biomedicines9081008/s1, Figure S1: Male RGS4-deficient mice show more pronounced hyperglycemia following oral glucose challenge, Figure S2: RGS4-deficient β-cells show normal subcellular distribution of docked and readily releasable insulin granules, Figure S3: RGS4 regulates glucose-stimulated insulin secretion via a somatostatin receptor-independent mechanism.
