# Supplementary material for: RGS4-Deficiency Alters Intracellular Calcium and PKA-Mediated Control of Insulin Secretion in Glucose-Stimulated Beta Islets
Source: Biomedicines. 2021 Aug 13;9(8):1008. doi: 10.3390/biomedicines9081008 (PMC8391461; doi:10.3390/biomedicines9081008)
Supplement: Supplementary file 1 [file biomedicines-09-01008-s001.zip › biomedicines-1300280-supplementary.pdf]

Supplementary Material

RGS4-Deficiency Alters Intracellular Calcium and PKA-Mediated Control of Insulin Secretion in Glucose-Stimulated Beta Islets

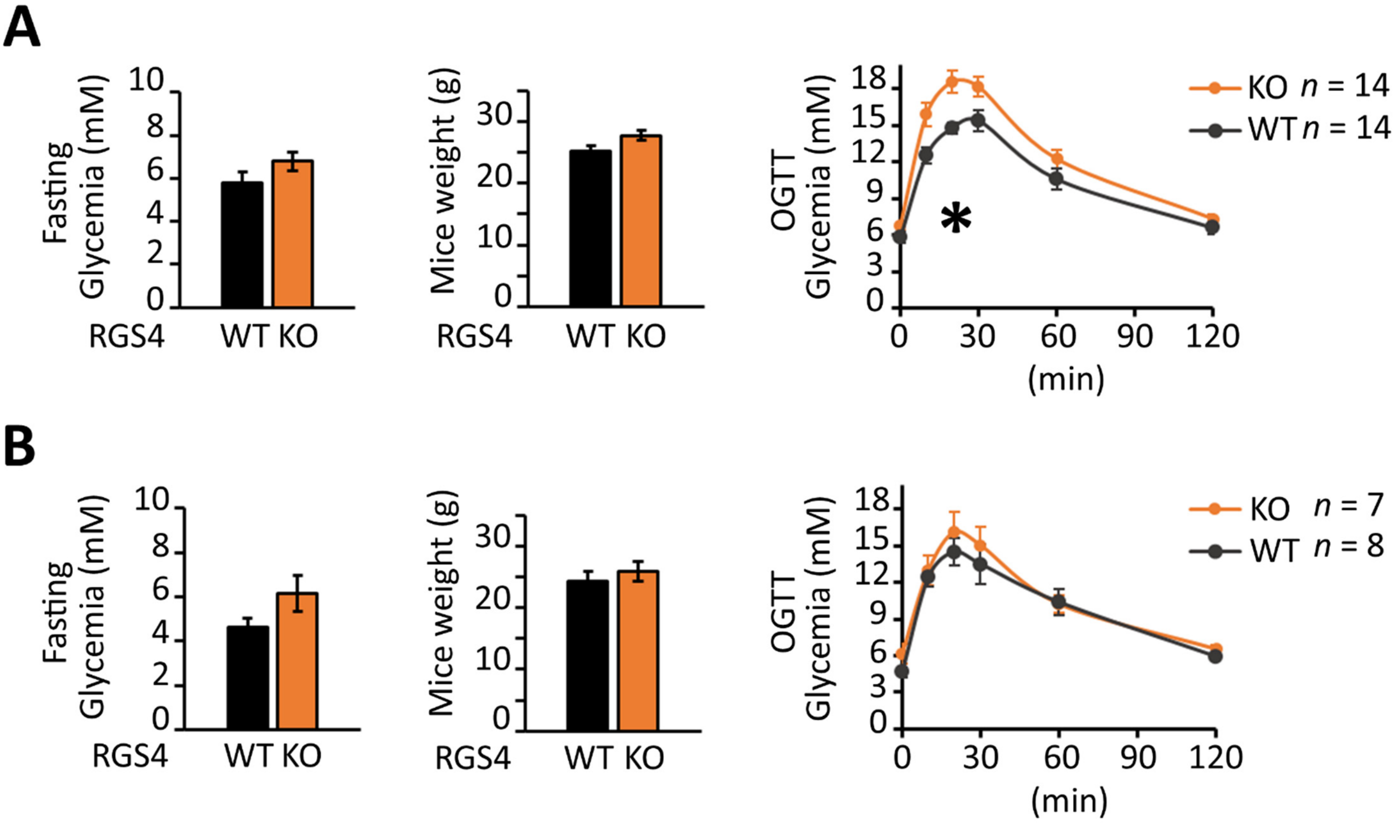

Figure S1. Male RGS4-deficient mice show more pronounced hyperglycemia following oral glucose challenge. Fasting glycemia levels, average weight, and glycemia kinetics following oral glucose challenge were recorded in male (**A**) and female (**B**) WT and RGS4-deficient mice. *p <0.05; [t-test for AUC was performed in panel **A** and **B**].

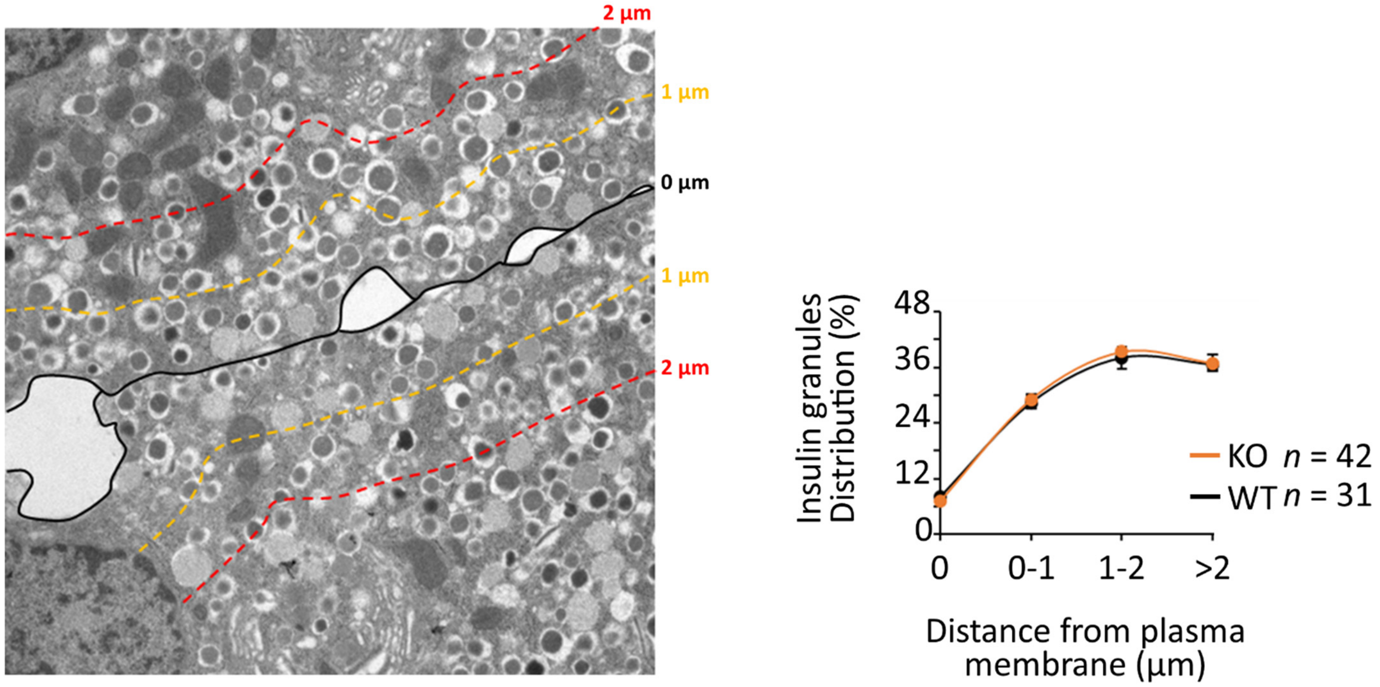

Figure S2. RGS4-deficient β-cells show normal subcellular distribution of docked and readily releasable insulin granules. The left panel shows a representative electron microscopy micrograph highlighting different subcellular compartments

according to their distance from the plasma membrane. The right panel shows the relative distribution of insulin granules in the different compartments for WT (*n*= 31 β-cells) and RGS4-KO (*n*= 42 β-cells).

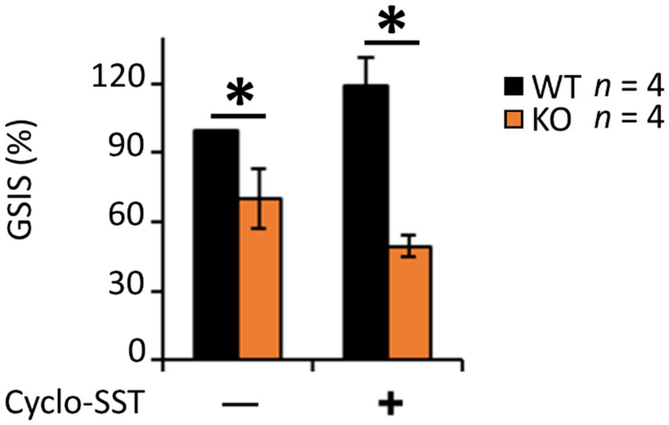

Figure S3. RGS4 regulates glucose-stimulated insulin secretion via a somatostatin receptor-independent mechanism. Glucose-stimulated insulin secretion (GSIS) was measured in isolated islets from WT and RGS4-KO mice following 30 min glucose challenge (20 mM) with and without the addition of the somatostatin receptor antagonist (cyclo-SST, 3 mM). Data are expressed as GSIS % relative to that for WT islets. **p* <0.05; NS, not significant [one-way ANOVA and Tukey's post-hoc test].
